# Supplementary figures and images for: Low energy multiple blue light-emitting diode light Irradiation promotes melanin synthesis and induces DNA damage in B16F10 melanoma cells
Source: PLoS One. 2023 Feb 2;18(2):e0281062. doi: 10.1371/journal.pone.0281062 (PMC9894472; doi:10.1371/journal.pone.0281062)

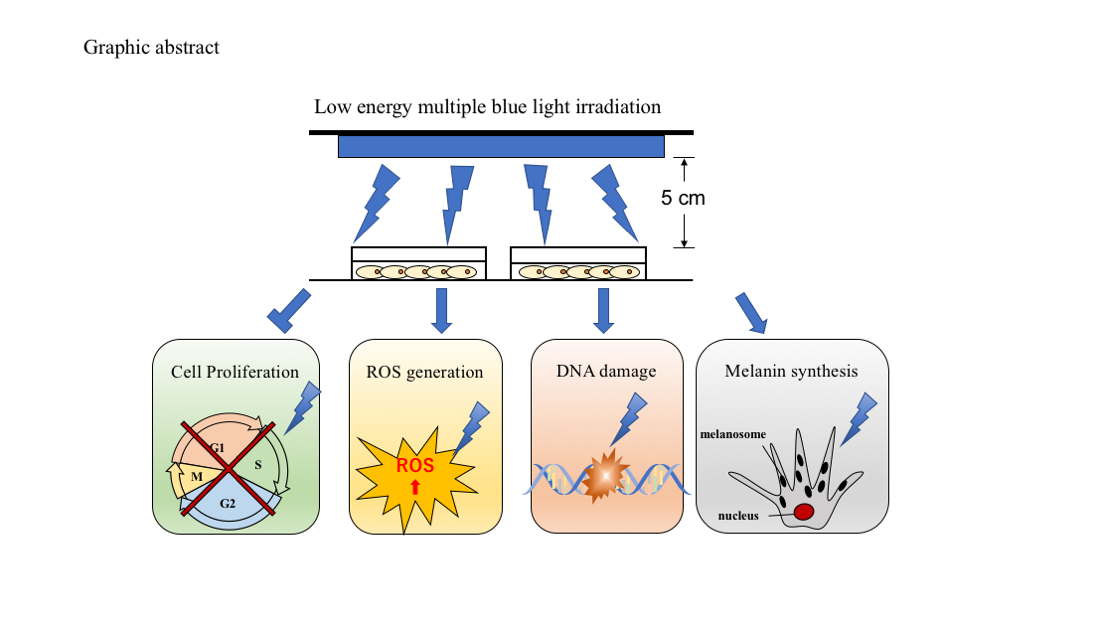

Supplement: S1 Graphic abstract — (TIF) [file pone.0281062.s002.tif]

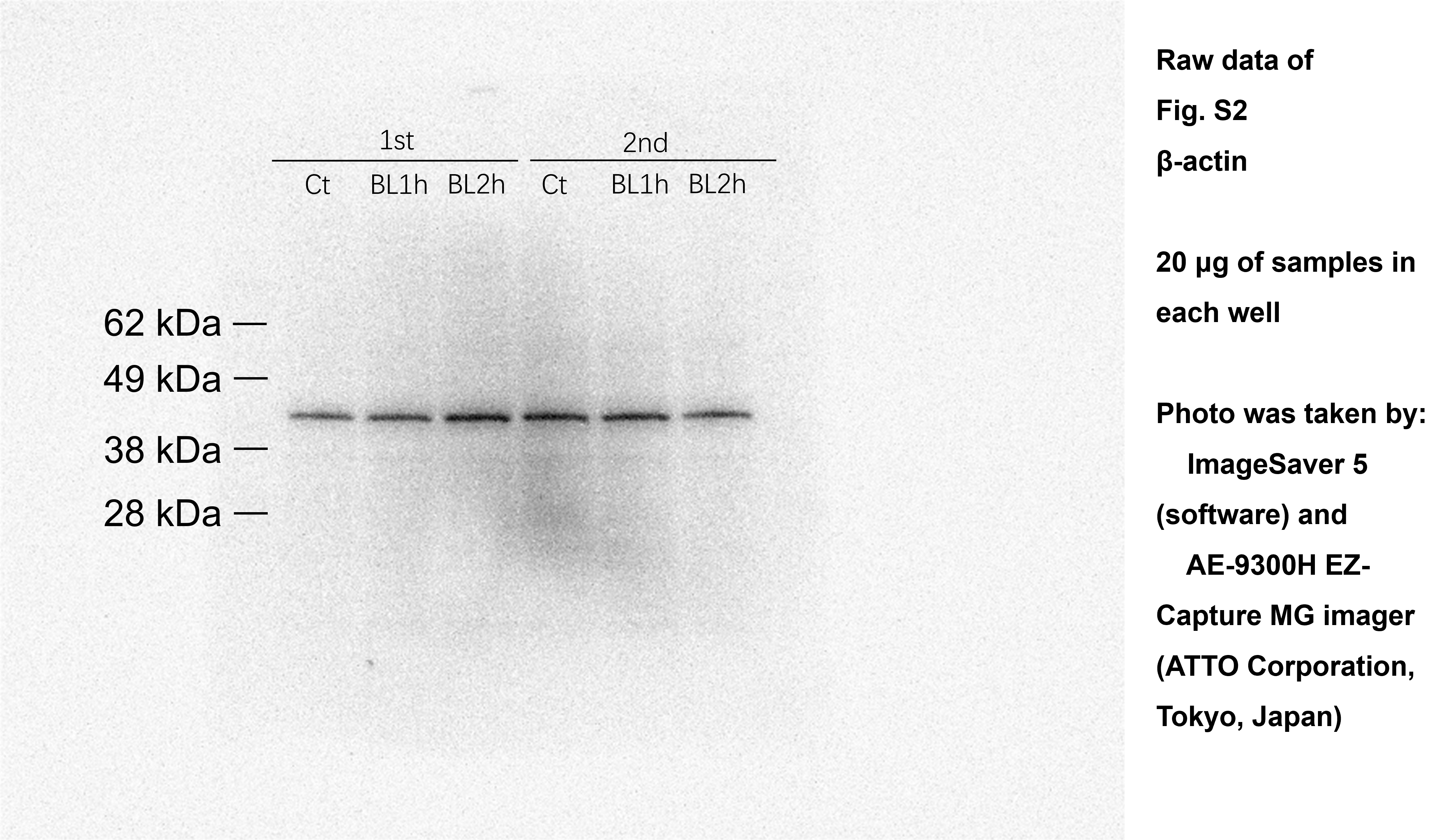

Supplement: S1 Raw images — (ZIP) [file pone.0281062.s004.zip › Figure S2_raw_images_β-actin.tif]

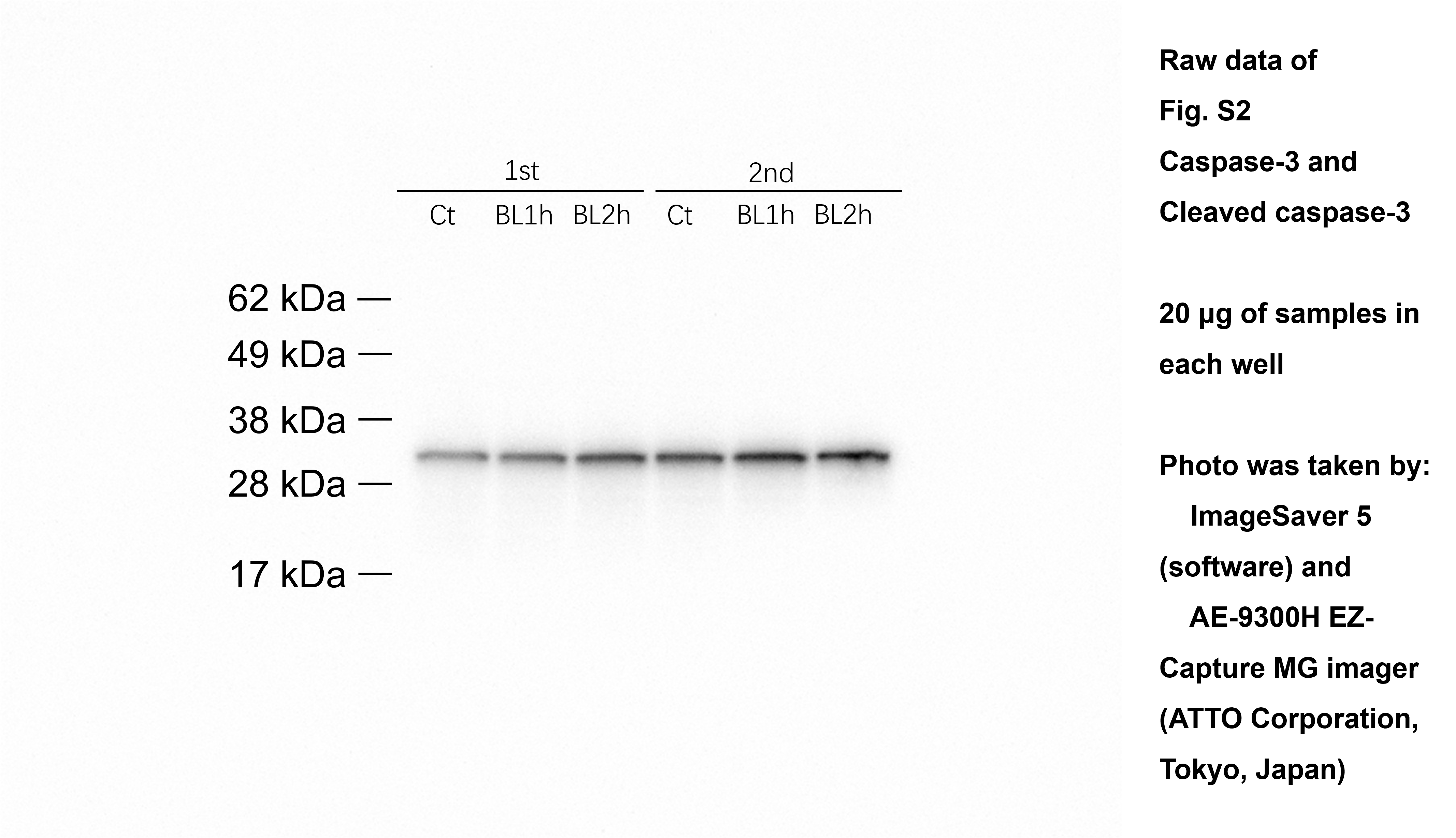

Supplement: S1 Raw images — (ZIP) [file pone.0281062.s004.zip › Figure S2_raw_images_caspase-3.tif]
