# Supplementary material for: Low energy multiple blue light-emitting diode light Irradiation promotes melanin synthesis and induces DNA damage in B16F10 melanoma cells
Source: PLoS One. 2023 Feb 2;18(2):e0281062. doi: 10.1371/journal.pone.0281062 (PMC9894472; doi:10.1371/journal.pone.0281062)

Supplemental figure 1

Cyclin mRNA level after irradiation

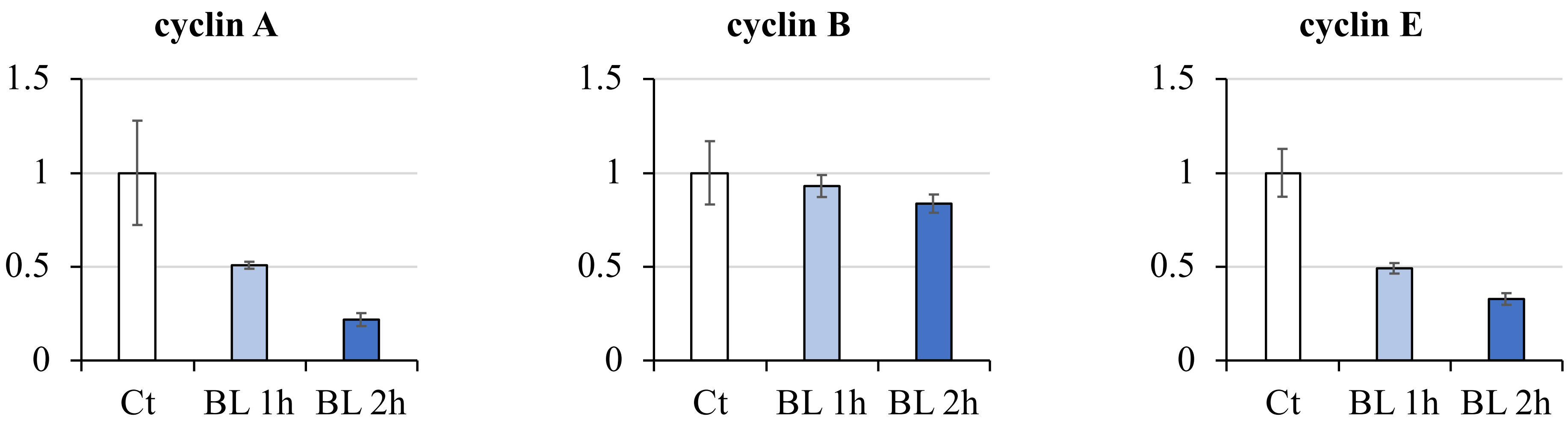

Supplemental figure 2

Cleaved - caspase 3 protein level after day 3 irradiation

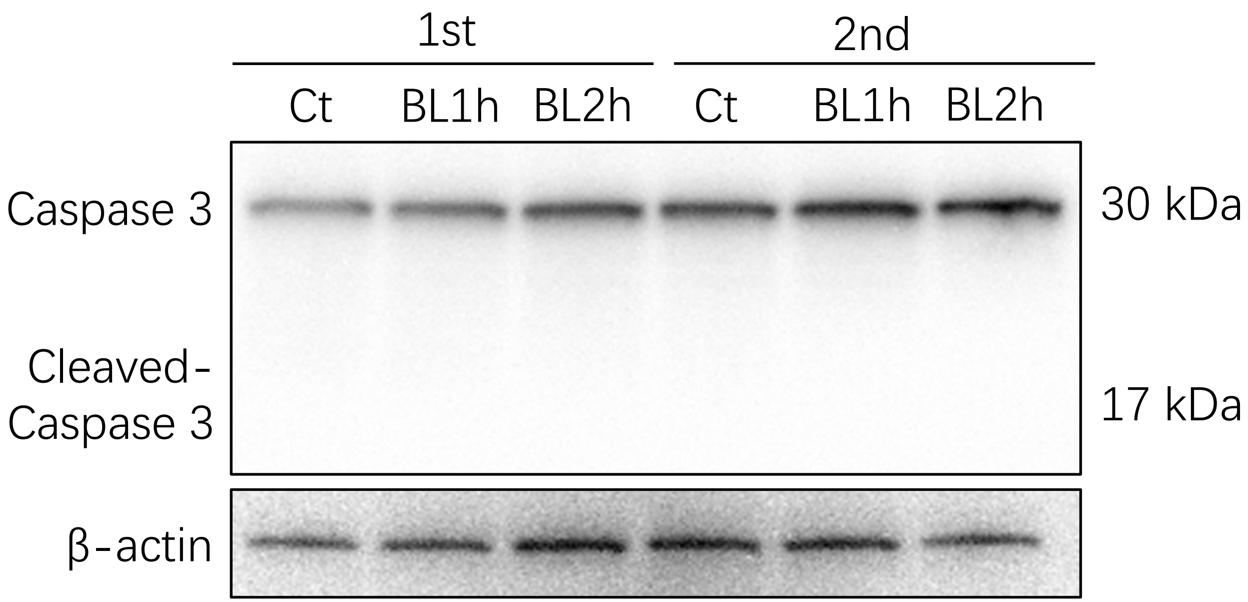

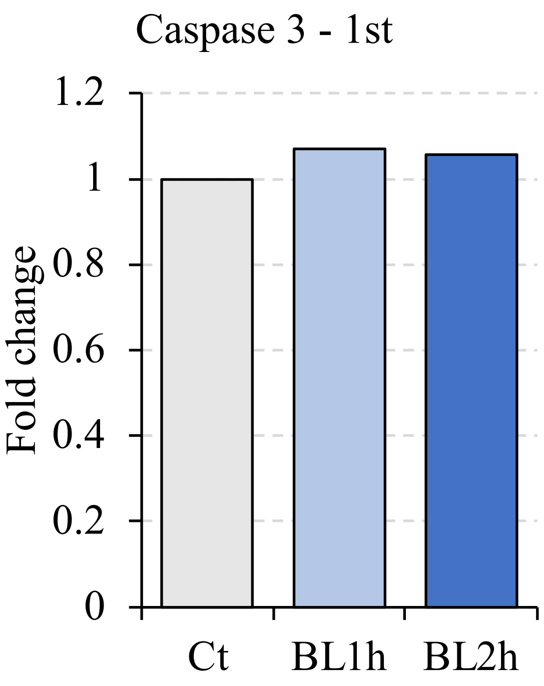

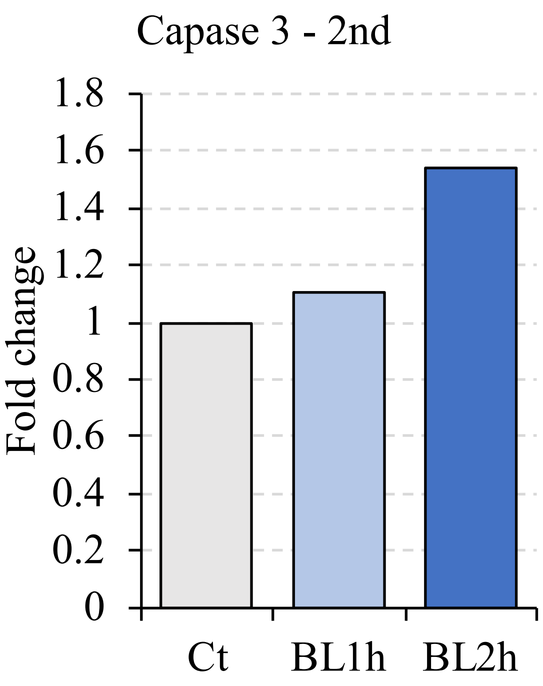

Supplemental figure 3

Clear field for ROS

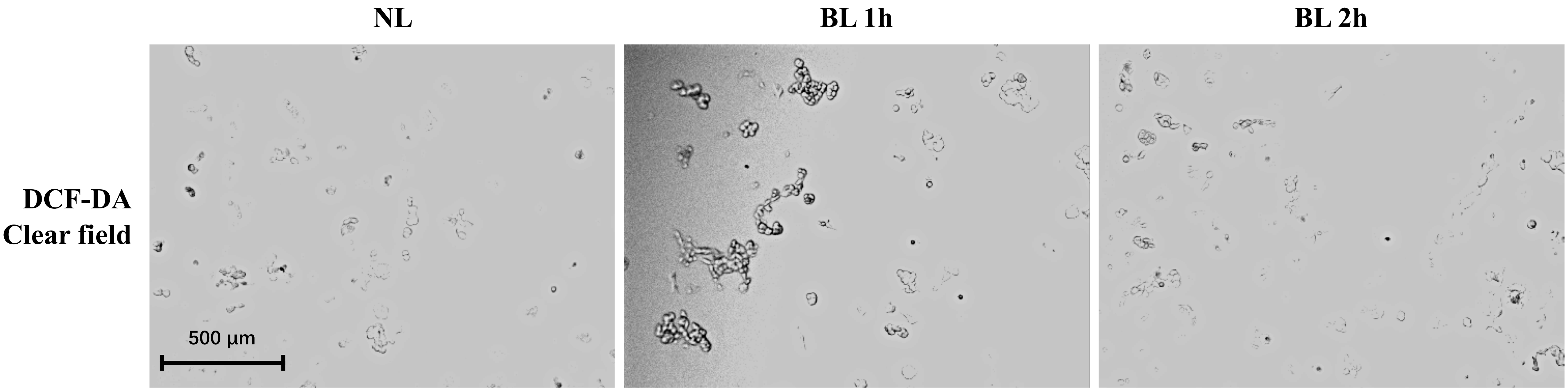

Supplemental figure 4

Clear field for DNA damage

NL **BL 1h** **BL 2h**

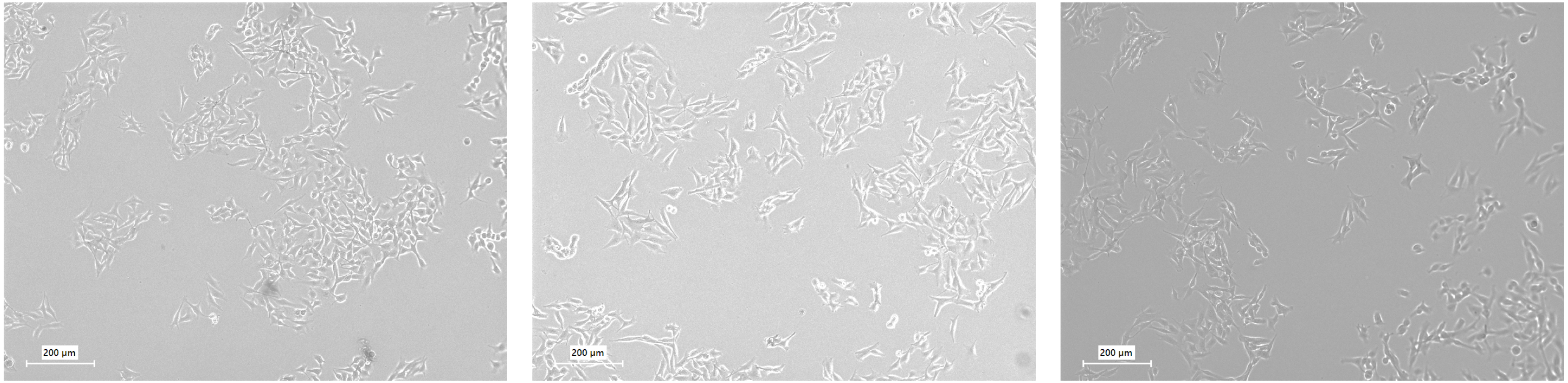

Supplement: S1 File — (PDF) [file pone.0281062.s003.pdf]
