# Supplementary material for: Low energy multiple blue light-emitting diode light Irradiation promotes melanin synthesis and induces DNA damage in B16F10 melanoma cells
Source: PLoS One. 2023 Feb 2;18(2):e0281062. doi: 10.1371/journal.pone.0281062 (PMC9894472; doi:10.1371/journal.pone.0281062)
Supplement: Attachment — Submitted filename: Response to reviewer-PONE-D-22-27769-rev2.docx [file pone.0281062.s006.docx]

**Dear editor:**

Thank you for giving us the opportunity to revise our manuscript for publication in PLOS ONE. We appreciate the time and effort that you and the reviewers dedicated to providing feedback on our manuscript and are grateful for the insightful comments on and valuable improvements to our paper.

We have incorporated most of the suggestions made by the reviewers. Those changes are highlighted in the manuscript. Please see below, in **red**, for a point-by-point response to the reviewers’ comments and concerns. All page numbers refer to the revised manuscript file with tracked changes.

**Reviewer #1:**

Title: Low Energy Multiple Blue light-emitting diode light Irradiation promotes melanin synthesis and induces DNA damage in B16F10 melanoma cells

Authors: Siqi ZHOU, Ryusuke YAMADA, Kazuichi Sakamoto

This paper provides important information that helps better understand the effect blue light has on cells. The abstract is clear and describes the background, goals, and results of the paper well.

Reply: Thank you very much.

As a general comment, the English grammar needs to be improved throughout the manuscript before the final submission. My specific comments are below:

**Introduction**

1. It may be helpful to provide further background and statistics on the prevalence of screen use in recent years, especially due to COVID. It would also be helpful to clearly specify what wavelength and energy of blue light is present in most daily use screens.

Reply: Thank you very much for your comments. We agree with your point of view very much, so that we have added the relevant descriptions about COVID and the usage of digital device in our introduction section as “A recent web-based survey indicated that approximately 93.8% of respondents reported that their digital device usage hours increased under the influence of COVID. In particular, the student population saw an increase of 5.18 ± 2.89 hours per day of screen-facing time due to e-learning.”, please refer to page 3 line 37 – 40.

For the wavelength and energy of blue light is present in most daily use screens, we also think it is very important condition. So we added the wavelength of our daily used digital device screens as “Therefore, it is important to investigate the effect of light from the LED screen (there is a peak of light intensity in the 400-500 nm range of the screen spectrum) on our bodies and cells” at page 3 line 42-43.

However, depending on the difference of devices, the duration (time) and the posture (distance) we used, the energy we got were different. Therefore, we are sorry that it is difficult to include a range of energy for daily use.

2. Authors discuss red light in the second paragraph of the introduction. It would be helpful to provide further background on the effect red light has on cellular proliferation, ROS, and DNA damage, as these are things this paper is studying.

Reply: Thank you very much. We have already provided the effect of red light on cell proliferation. We also searched the previous studies about the effect of red light on oxidative stress and DNA damage. These contents have been inserted into the introduction section as “And red light exposure does not cause oxidative stress and DNA damage”, please refer to page 3 line 47.

**Methods**

1. Please specific the light irradiation protocol (ex/ how many hours of irradiation per day) in the “Light irradiation” section of the introduction.

Reply: Thank you very much. We have revised the irradiation protocol to “For the 3-day experiment, the cells were exposed to blue light for 0, 1, and 2 hours per day and then returned back to the incubator (dark). After the irradiation of day 3, the cells were returned to the dark incubator for 24 hours before subsequent assays.”, please refer to page 6 line 99-101.

2. What plating density was used for the light irradiation?

Reply: Thank you very much. The plating density of cells was 1.0 × 10^5^ cells/ well in 60 mm dish, 3.0 × 10^3^ cells/well in 24-well-plates, or 1.0 × 10^3^ cells/well in 96-well-plates. We have mentioned this in the method section cell and cell culture (in page 5 line 90). We also checked and revised the description in each method of assay as:

“Cells were cultured in 24-well-plates as a density of 3.0 × 10^3^ cells/well” in the method of MTT assay,

“Cells were cultured in 24-well-plates as a density of 3.0 × 10^3^ cells/well.” in the method of Trypan blue assay and proliferation assay,

“Cells were prepared in a Nunc black wall clear flat bottom 96 well plate (Thermo Fisher Scientific, USA) as a density of 1.0 × 10^3^ cells/well.” in the method of apoptosis assay and ROS generation assay.

“1.0 × 10^5^ cells were seeded into 6 cm dishes until confluent.” in the method of wound healing assay.

“Cells for melanin content were prepared in a 6-well-plate as a density of 5.0 x 10^4^ cells/well” in the method of melanin content assay.

“Cells were prepared in 6 cm dishes as a density of 1.0 × 10^5^ cells/dish” in the method of real time PCR

3. Please specify how long after light irradiation each assay was performed.

Reply: Thank you very much. We have checked and revised the time after irradiation for the method of each assay.

“The enzymatic activity was measured daily after the irradiation 24 hours.” in the method of MTT assay,

“The green fluorescence (Ex/Em = 490/525 nm) for apotosis cells and the blue (Ex/Em = 405/450 nm) for alive cells were stained after the day 3 irradiation 24 hours” in the method of apoptosis assay,

“Intracellular reactive oxygen species (ROS) were immediately measured after the irradiation with a DCFDA/H2DCFDA Cellular ROS assay kit” in the method of ROS generation,

“After day 3 irradiation, cells were returned to the dark incubator for 24 hours and then collected with trypsin,” in the method of melanin content assay,

“mRNA (immediately and 3 hours after blue light irradiation) was extracted using RNA iso Plus” in the method of Real time PCR.

**Discussion**

1. What is the significance of the fact that blue light inhibited cellular proliferation, but did not cause apoptosis? (page 14)

Reply: Thank you very much for your comment. Unfortunately, according to our results, it was not enough to explain the significance of these phenomenon very well. We thought this will be a very interesting topic in the future. We added some statement in our discussion part to show our inadequacy.” Longer wavelength lower energy blue light irradiation inhibited cell proliferation without inducing apoptosis. This is a very interesting phenomenon. To cancer treatment, it means that it is possible to inhibit cancer growth without killing the cells. While further experiments are still necessary to verify this conjecture.” Please refer to page 15 line 301-305.

2. Perhaps more clearly discuss why the MTT assay showed reduced cell enzymatic activity, but the trypan blue assay shows no impact on cell survival in the discussion.

Reply: Thank you very much for your comment. We discussed this point in discussion section more clearly as “Initially, we found the blue light irradiation decreased the cell enzymatic activity by MTT assay but didn’t affect the cell survival rate by trypan blue assay. Since the MTT results were based on both cell number and enzymatic activity, which a decreased cell number was also found. It was difficult to determine whether the decrease in MTT was due to a decrease in cell number or a decrease in cell enzymatic activity. Therefore, we further performed trypan blue and cell proliferation assays. Intermittent, multiple irradiations didn’t affect the cell survival rate but significantly reduced cell proliferation. These results indicated that the decreased enzymatic activity by blue light due to the reduced cell proliferation rather than cytotoxicity.”, please refer to page 15 line 287-294.

3. What is the significance of blue light inhibiting cell migration? (page 15)

Reply: Thank you very much for your comment. As B16F10 melanoma cells were metastatic cells, the inhibition for migration maybe can give us some idea about blue light exposure inhibits the melanoma metastasis (benefit). The inhibition effect on cells migration also gave us ideas about blue light exposure slow down the wound healing (harm). Compared to other previous studies, blue light usually inhibits proliferation and metastasis. Therefore, we believe that our result of migration inhibition effect is still mainly indicative of the harmful effects of blue light. Therefore, we summarized this migration inhibition effect, together with DNA damage, proliferation inhibition as a weak phototoxicity in conclusion. “low energy density blue light has a weak phototoxicity, repeated exposure will also cause cellular damage and regulate cellular function” please refer to page 18 line 370.

4. What do authors mean when they state “intracellular ROS and DNA damage immediately occurred after blue light irradiation, and there was no significant change after three days irradiation? (page 16 line 327). In the methods and results, authors only discussed that immediately after blue light irradiation, ROS production and DMA damage was increased.

Reply: Thank you very much for your comment. At the beginning of ROS and DNA damage assay, we also detected the cells after 3 days treatment (the same condition as MTT, trypan blue, proliferation, and apoptosis). But we didn’t detect some significant change. We thought the reason was that we gave enough time to cells recovery. Therefore, we further directly detect the change of intracellular ROS and DNA damage immediately after the irradiation of blue light. So these results means the ROS generation and DNA damage occurred immediately after the irradiation, which was not an irreversible effect. This result is our prior experimental result is not presented in this paper, so we changed this sentence to " there was no significant change after three days irradiation in our previous study " please refer to page 17 line 350.

5. In the abstract, authors state “low energy density blue light multiple exposure is still harmful to our cells even though the effect is lower than short wavelength high energy blue light.” Please cite evidence of this and discuss in the discussion.

Reply: Thank you very much for your comment. We think your comments are very helpful. We have mentioned the harmful effects of short wavelengths on cells from previous studies in our discussion, but we did not specifically compare the difference between long and short wavelengths. We thought that short wavelength led to apoptosis and longer wavelength didn’t induce apoptosis, is the relatively lower harmful effect. However, this idea is indeed arbitrary. So that we removed our arbitrary description in abstract. “Together, these results indicate that longer wavelength and low energy density blue light multiple exposure is still harmful to our cells.” please refer to page 2 line 26 .

Figures

1. Please define all abbreviations used in figures in the key (ex/ NL, B1h, B2h)

Reply: Thank you very much for your comment. We added the abbreviation in the legends of figures.

“NL: non-light treatment; BL: blue light irradiation; B1h, B2h: blue light irradiation 1, 2 hours.” in figure 1,

“NL: non-light treatment, BL: blue light irradiation.” in figure 2,

“NL: non-light treatment, BL: blue light irradiation.” in figure 3,

“NL: non-light treatment, BL: blue light irradiation, tyr: tyrosinase.” in figure 4.
